# Inconsistent clinical outcomes following afatinib treatment in NSCLC patients harboring uncommon epidermal growth factor receptor mutation

**DOI:** 10.3389/fonc.2022.999606

**Published:** 2022-11-08

**Authors:** Wei Dong, Congjie Wang, Chunsheng Wang, Kewei Zhao, Zhao Ma, Shanliang Hu

**Affiliations:** ^1^ Department of Radiation Oncology, Yantai Yuhuangding Hospital, Yantai, Shandong, China; ^2^ Department of Pulmonary and Critical Care Medicine, Yantai Yuhuangding Hospital, Yantai, Shandong, China; ^3^ Department of Radiation and Medical Oncology, Zhongnan Hospital of Wuhan University, Wuhan, Hubei, China; ^4^ Cancer Center, Union Hospital, Tongji Medical College, Huazhong University of Science and Technology, Wuhan, Hubei, China

**Keywords:** afatinib, uncommon, EGFR, efficacy, prognosis, NSCLC

## Abstract

**Background:**

Uncommon epidermal growth factor receptor (EGFR) mutations consist of a heterogeneous population of molecular alterations, and the available clinical data on the outcomes of patients with non-small-cell lung cancer (NSCLC) harboring uncommon EGFR mutations following afatinib treatment are limited. The purpose of this pooled analysis was to investigate the clinicopathological features of patients with uncommon EGFR mutations (um-EGFRms) along with their treatment response and survival outcomes following afatinib treatment.

**Methods:**

We performed a literature search in the NCBI PubMed database to identify relevant articles and conducted this pooled analysis based on 70 studies. The relationships between patient clinical characteristics, EGFR mutation type and the response to afatinib treatment were analyzed using univariate chi-square analysis, and survival analysis was performed using the Kaplan–Meier method.

**Results:**

Data from a total of 99 patients were included in the pooled analysis. The objective response rate (ORR) to treatment with afatinib was53.5%, with a median progression-free survival (mPFS) of 9.0 months. For patients administered first-line afatinib treatment, the ORR and median PFS were 73.5% and 15.6 months, respectively, which were both superior to those of patients treated with second- or later-line treatments (ORR:37.0%, p < 0.001; mPFS: 6.0months, p = 0.001). Moreover, patients with a single um-EGFRm were more likely to have a favorable response and prognosis benefit after treatment with afatinib than patients with multiple one (ORR: 63.3% vs 38.5%, p=0.017; mPFS: 15.6 months vs 6.0 months,p=0.010). Moreover, single um-EGFRm were independent predictive factors for better treatment response and superior PFS. Subgroup analysis indicated that patients harboring major um-EGFRms (i.e., L861Q, G719X, and S768I) exhibited the best treatment responses and prognoses (ORR: 74.1%, mPFS: 15.6 months), by contrast, patients harboring multiple um-EGFRms comprising 19del/L858R had the worst treatment responses and prognoses (ORR: 23.5%, mPFS: 5.6months).

**Conclusions:**

Patients with um-EGFRms exhibit favorable but inconsistent responses and survival outcomes following afatinib treatment, which closely related to the mutation pattern and cooccurring partner mutant genes. Administering afatinib for the treatment of patients with um-EGFRm might be considered an effective treatment option in some circumstances, but this recommendation requires further clinical studies for verification.

## Introduction

Epidermal growth factor receptor (EGFR) mutations play an important role in the pathogenesis of non-small-cell lung cancer (NSCLC) and are one of the main oncogenic drivers of NSCLC. The frequency of EGFR mutations in NSCLC patients in the Caucasian population is 10%-20%, while it is as high as 30%-60% in the Asian population (1–3). The most prevalent EGFR mutation is exon 19 deletion (19del), followed by point mutation L858R in exon 21 (3). Both are considered to be common and sensitive mutations of EGFR, accounting for 80-90% of mutations in the EGFR gene (3–5). A number of clinical studies have confirmed that, compared with traditional chemotherapy, treatment with EGFR-tyrosine kinase inhibitors (EGFR-TKIs) results in an objective response rate (ORR) as high as 70%-80%, a median progression-free survival (mPFS) of 9.6 months to 18.9 months, and an overall survival (OS) of 21.6 months to 34.1 months (6–12). Nowadays, EGFR-TKIs have become the first-line standard treatment for advanced NSCLC patients with EGFR-sensitive mutations. Additionally, other types of EGFR mutations, such as insertions in exon 20 (20 ins), G719X in exon 18, S768I in exon 20, and L861Q in exon 21 were also found, which are called uncommon EGFR mutation (um-EGFRm), accounting for approximately 10% to 15% of EGFR mutations (13–16). Since patients with um-EGFRms are relatively insensitive to treatment with EGFR-TKIs, which may have a negative impact on research results, most clinical trials investigating the efficacy of EGFR-TKIs do not include patients with this mutation type (13–16). Due to small sample size and high heterogeneity, the efficacy of EGFR-TKIs for patients with um-EGFRms is still unclear. With the rapid development of genetic testing technology, the detection rate of um-EGFRms will continue to increase, and it is of great significance to better understand the sensitivity, efficacy and prognosis of these patients to various TKIs.

It was reported that afatinib, an irreversible ErbB family blocker, is more effective than first-generation TKIs in treating patients with um-EGFRms (14, 17–25). The Food and Drug Administration (FDA) has approved afatinib for the treatment of metastatic NSCLC patients with major um-EGFRms (G719X, S768I, and L861Q). However, unlike common EGFR mutations, which only include two types, um-EGFRms are a class of highly heterogeneous mutations. Due to the low frequency of um-EGFRms and the uncertain efficacy of afatinib, the number of patients receiving afatinib in clinical practice was relatively small (13, 14). Thus, we conducted this pooled analysis to explore the clinical characteristics of patients with um-EGFRms, as well as the efficacy and prognosis following treatment of afatinib, so as to provide a reference for clinicians who formulate treatment plans for patients with rare EGFR mutations.

## Methods

### Search strategy

We performed a literature search in the NCBI PubMed database to identify all the relevant articles without language restriction (the last search update was June 15, 2021). The following search strategy were used: ((afatinib[title/abstract]) and ((EGFR [title/abstract]) or epidermal growth factor receptor[title/abstract])) and ((NSCLC [title/abstract]) or non-small cell lung cancer[title/abstract]). We also manually checked the reference lists of all related articles to add to the research.

### Study eligibility

Two authors independently screened the titles and abstracts of the studies from the search results, and a second screening of the full-text articles was performed. If these two authors failed to reach a consensus, a third investigator was consulted to resolve any disagreements and to reach a consensus on all items. Articles were included if they met the following inclusion criteria: 1) studies focusing on patients with non-small-cell lung cancer; 2) studies in which all patients harbored non-ex20ins, uncommon mutations in EGFR (without restrictions in the method and the biological source for mutation test); 3) patients received afatinib in any treatment line; 4) studies indicating treatment response to afatinib; and 5) studies that reported the PFS of patients.

### Study objective

The following data of patients were collected: age, gender, ethnicity, smoking history, tumor stage, mutation type, response to afatinib (objective response (OR) was defined as CR+PR), and PFS. Tumor response was defined as complete response (CR), partial response (PR), stable disease (SD) or progressive disease (PD) based on Response Evaluation Criteria in Solid Tumors. Objective response (OR) was defined as CR+PR. The primary objective of this study is the clinical outcome of patients applying afatinib treatment, which includes objective response rate (ORR) and progression free survival.

### Exploratory analysis

Due to the relatively high incidence of L861Q, G719X, and S768I, they are referred to as major uncommon mutations. Considering that uncommon EGFR-mutant NSCLC is a genetically heterogeneous disease, and the FDA has approved the use of afatinib for the treatment of patients with um-EGFRms of L861Q, G719X, and S768I. We have great interested in the outcomes of afatinib in patients with different types of um-EGFRm. Therefore, we conducted subgroup analyses of afatinib efficacy and survival in patients with different um-EGFRm patterns: Group A for major um-EGFRms (i.e., G719X, S768I, and L861Q), Group B for other single um-EGFRms, Group C for multiple EGFR mutations that contains 19del/L858R, and Group D for multiple EGFR mutations that without 19del/L858R.

### Statistical analysis

Fisher’s exact or chi-squared tests were used to assess the associations between clinical parameters and afatinib efficacy. The Kaplan–Meier method and the log-rank test were used to analyze the association of clinical parameters with PFS, and the associated 95% CIs were calculated. Multivariate analysis was performed using logistic regression models and Cox proportional hazards models to assess the simultaneous effects of prognostic factors on efficacy and survival. The analyses were performed with SPSS 22.0 program (SPSS Inc, Chicago, IL, USA), a two-sided p-value less than 0.05 was considered statistical significance.

## Results

### Search results

**Figure 1:**
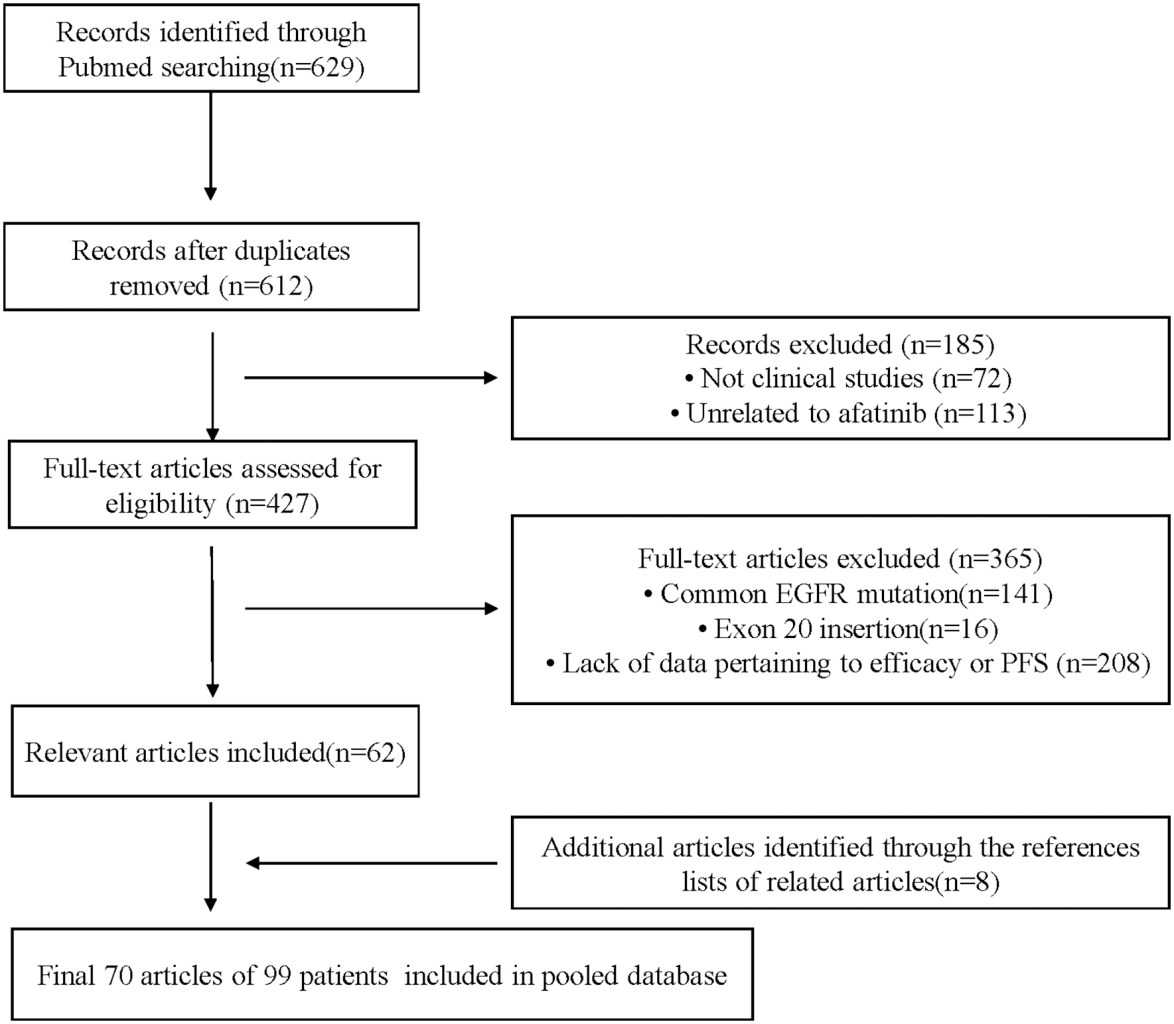
Flow chart of study process.

The flow chart of the study selection process is shown in **Figure 1**. A total of 679 potentially relevant articles were identified from the PubMed database. Two investigators individually screened the titles/abstracts and full texts and then extracted data separately. Finally, 70 articles were included in the pooled analysis (**Supplement Table 1**).

### Patient characteristics

**Figure 2:**
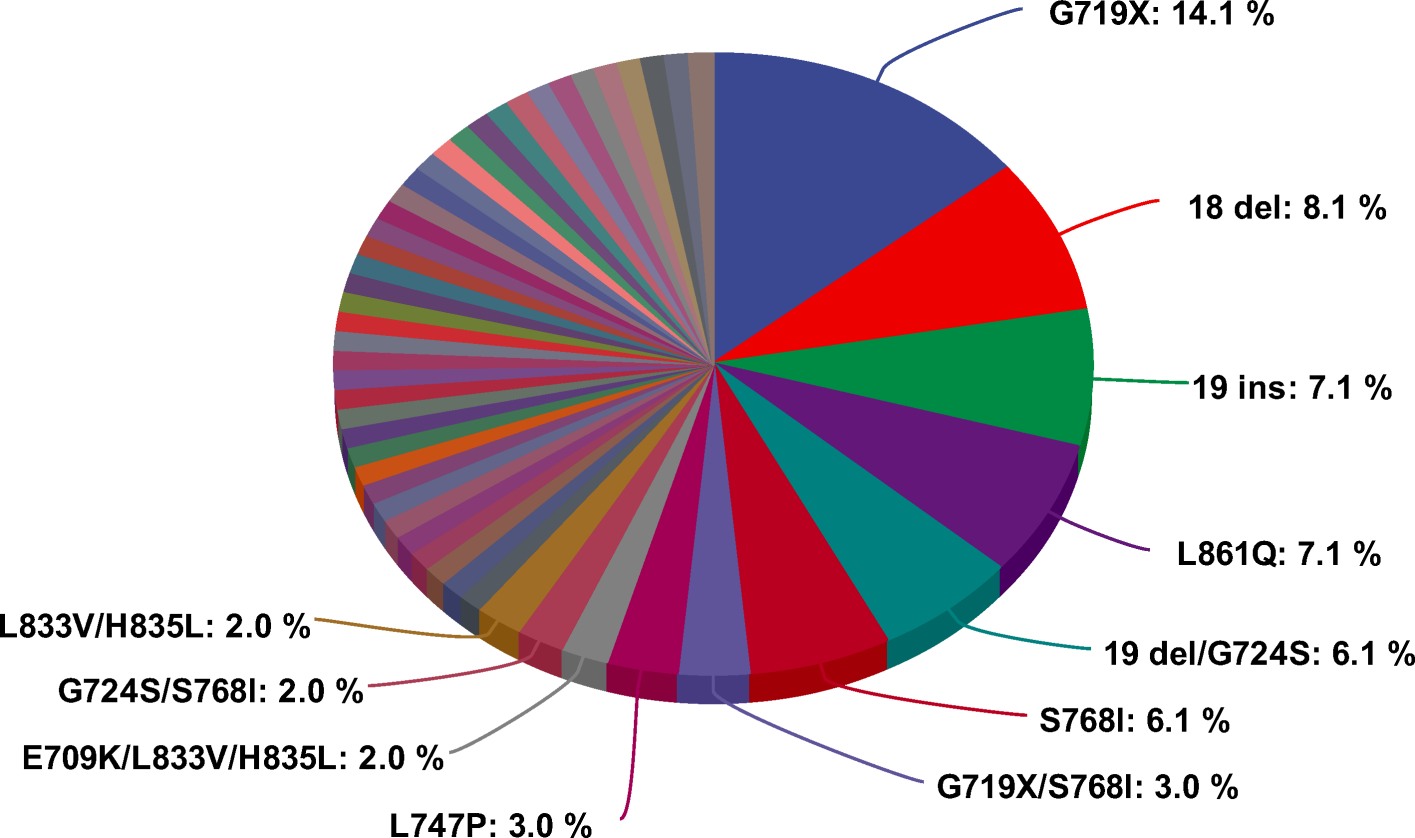
Composition of un-common EGFR mutations (n=99).

Data from a total of 99 patients were included in the pooled analysis, with a median age of 58 years and a range of 34 to 84 years. The sex distribution was basically balanced (53 males, 53.5%; 46 females,46.5%), and most were Asian patients (Asian 66, 66.7%; non-Asian 33, 33.3%). Nearly one-third of patients had a history of smoking (60, 60.6%), and most patients had stage IV disease (94, 94.9%). In terms of the mutation type, two-thirds of patients had a single um-EGFRm. The baseline characteristics of the patients are detailed in **Table 1**. In these 99 patients, there were a total of 50 kinds of um-EGFRms. The top six EGFR mutation types were G719X, 18 del, 19 ins, L861Q, 19del/G724S, and S768I, with 14 cases (14.1%), 8 cases (8.1%), 7 cases (7.1%), 7 cases (7.1%), 6 cases (6.1%), and 6 cases (6.1%), respectively (**Figure 2**; **Supplement Table 2**).

**Table 1:** Baseline characteristics.

Characteristics	No. (n=99)	percentage
Age		
<60	54	54.5%
≥60	45	45.5%
Gender		
Male	53	53.5%
Female	46	46.5%
Ethnicity		
Asian	66	66.7%
Non-Asian	33	33.3%
Smoking		
Yes	39	39.4%
No	60	60.6%
Stage		
I-III	5	5.1%
IV	94	94.9%
EGFR test		
DNA Sanger sequencing	17	17.2%
NGS	35	35.3%
PCR	10	10.1%
ARMS	6	6.1%
NA	30	30.3%
Mutation number		
Single	60	60.6%
Multiple	39	39.4%
Afatinib lines		
1 Line	45	45.5%
≥2 Line	54	54.5%
Response to TKI		
CR	0	0.0%
PR	53	53.5%
SD	33	33.3%
PD	13	13.1%
PFS		
median	9.0 months	–

NGS, Next-generation sequencing; PCR, polymerase chain reaction; ARMS, amplification refractory mutation system; NA, not available; CR, complete response; PR, partial response; SD, stable disease; PD, progressive disease; PFS, progression-free survival.

### Clinical outcomes

**Figure 3:**
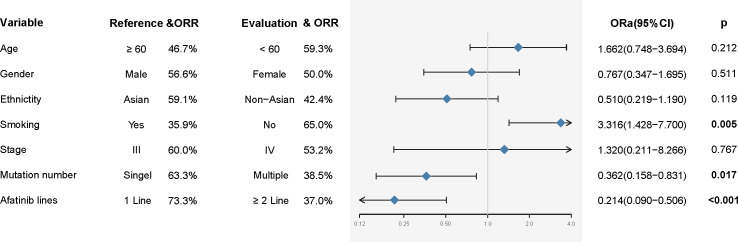
Univariate analysis for treatment response. ORR, Objective response rate; ORa, Odds ratio.

After treatment with afatinib, of the 99 patients included, none of the patient had a complete response (0.0%), 53 patients had a partial response (53.5%), and 33 patients had stable disease (33.3&); the other 13 patients experienced disease progression (13.1%) (**Table 1**). Overall, the objective response rate to the treatment with afatinib was53.5%. In univariate analysis, we found that patients receiving first-line afatinib had an ORR of73.3%, which was significantly better than that of patients receiving second- or later-line therapy (p<0.001). In addition, we found that there was a significant correlation between smoking and treatment efficacy and that patients without a history of smoking showed a significantly superior tumor response than those who smoked (ORR: 65.0% vs 35.9%, HR: 3.316, 95% CI: 1.428-7.700, p=0.005). Moreover, there is a trend favoring the patients with a single um-EGFRm, the difference in treatment efficacy between patients with single and multiple um-EGFRms was statistically significant (ORR: 63.3% vs 38.5%, HR: 0.362, 95% CI: 0.158-0.831, p=0.017). Other factors (e.g., age, sex, ethnicity, stage) did not show any correlation with efficacy (**Figure 3**). Further multivariate analysis suggested that not smoking (p=0.020), single um-EGFRm (p=0.040), and first-line treatment (p=0.004) were independent predictive factors for better treatment response (**Table 2**).

**Table 2:** Multivariate analysis for efficacy and PFS.

Variable	Multivariate analysis for efficacy			Multivariate analysis for PFS		
	ORa	95%CI	p	HR	95%CI	p
Smoking						
No	1.00			1.00		
Yes	0.333	0.132-0.838	**0.020**	1.543	0.932-2.555	0.092
Mutation number						
Single	1.00			1.00		
Multiple	0.379	0.151-0.956	**0.040**	1.866	1.128-3.088	**0.015**
Afatinib lines						
1 Line	1.00			1.00		
≥2 Line	0.262	0.106-0.646	**0.004**	1.800	1.057-3.064	**0.031**
TKI response						
OR	–	–	–	1.00		
Non-OR	–	–	–	2.554	1.525-4.277	**<0.001**

OR, objective response; ORa, odds ratio; HR, hazard ratio. P values < 0.05 are highlighted in bold.

**Figure 4:**
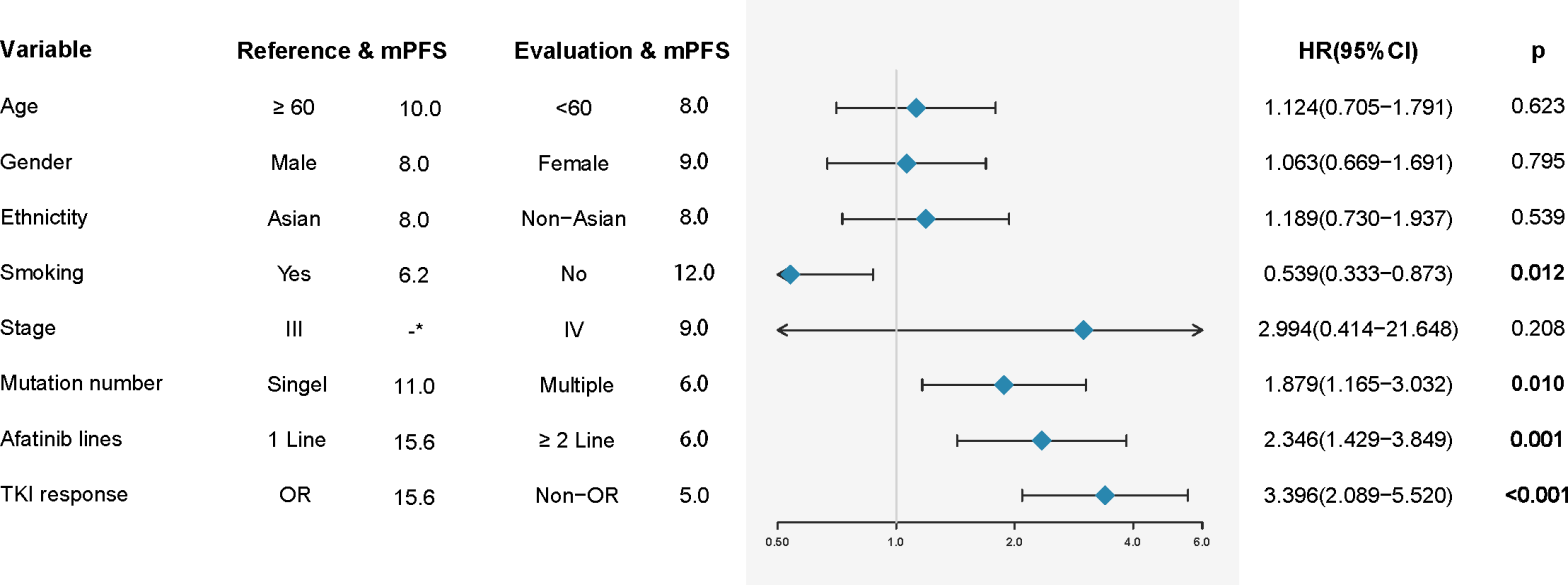
Univariate analysis for progression free survival (PFS). NA,s Not available.

**Figure 5:**
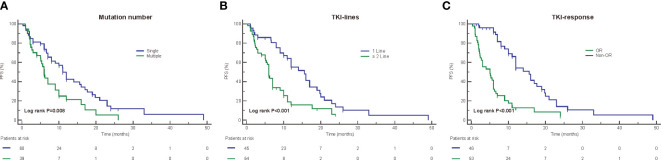
Kaplan-Meier curves for progression free survival (PFS) according to mutation numbers **(A)**, TKI-lines **(B)**, and TKI-response **(C)**.

In the overall population, the median progression-free survival time was 9.0 months (**Table 1**). The mPFS in patients receiving first-line treatment was 15.6 months, which was significantly better than that in patients who received second- or later-line treatment, which was 6.0 months (HR2.346, 95% CI: 1.429-3.849, p=0.001) (**Figures 4**, **5B**). In addition, for patients with a treatment response of OR (p<0.001) (**Figure 5C**), as well as no smoking history(p=0.012), the PFS was also longer. The Kaplan–Meier curves showed a trend that patients with a single um-EGFRm had longer PFS than patients with multiple um-EGFRms (**Figure 5A**), the difference was statistically significant(p=0.008). Subsequently, the results of the Cox proportional hazards model showed that the administration of first-line treatment, the objective response to treatment, and with single um-EGFRm were independent prognostic factors for longer PFS (**Table 2**).

### Subgroup analysis

**Figure 6:**
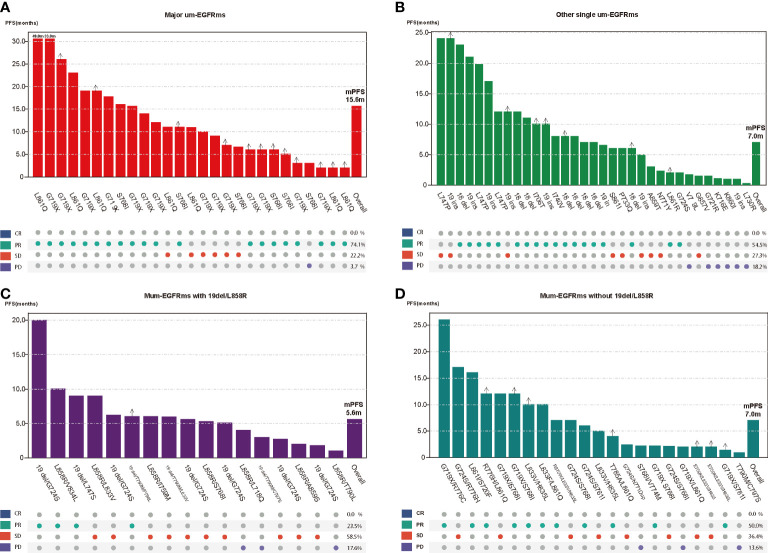
Tumor response and progression free survival (PFS) of each individual patient as well as overall tumor response rate and median PFS for each group (**A**: Major ucm-EGFRms; **B**: Other single ucm-EGFRm; **C**: multiple ucm-EGFRms that with 19del/L858; **D**: multiple ucm-EGFRms that without 19del/L858R).

**Figure 7:**
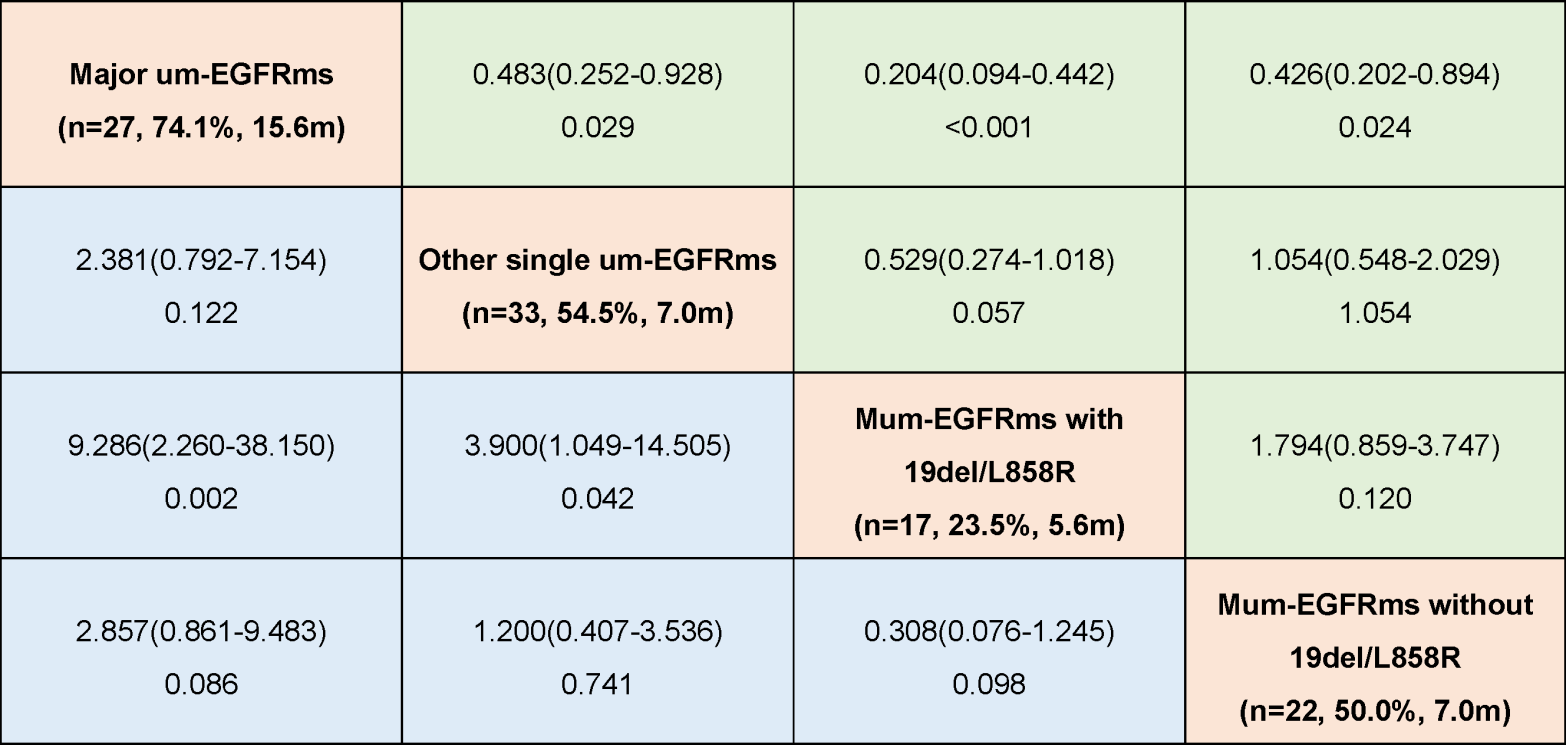
Odds ratio (ORa) with 95% CI for objective response rate (ORR) (blue)and hazard ratio (HR)with 95% CI for progression free survival (PFS)(green) in subgroup analysis according to mutation patterns (OR and HR was set by column versus row).

**Figure 8:**
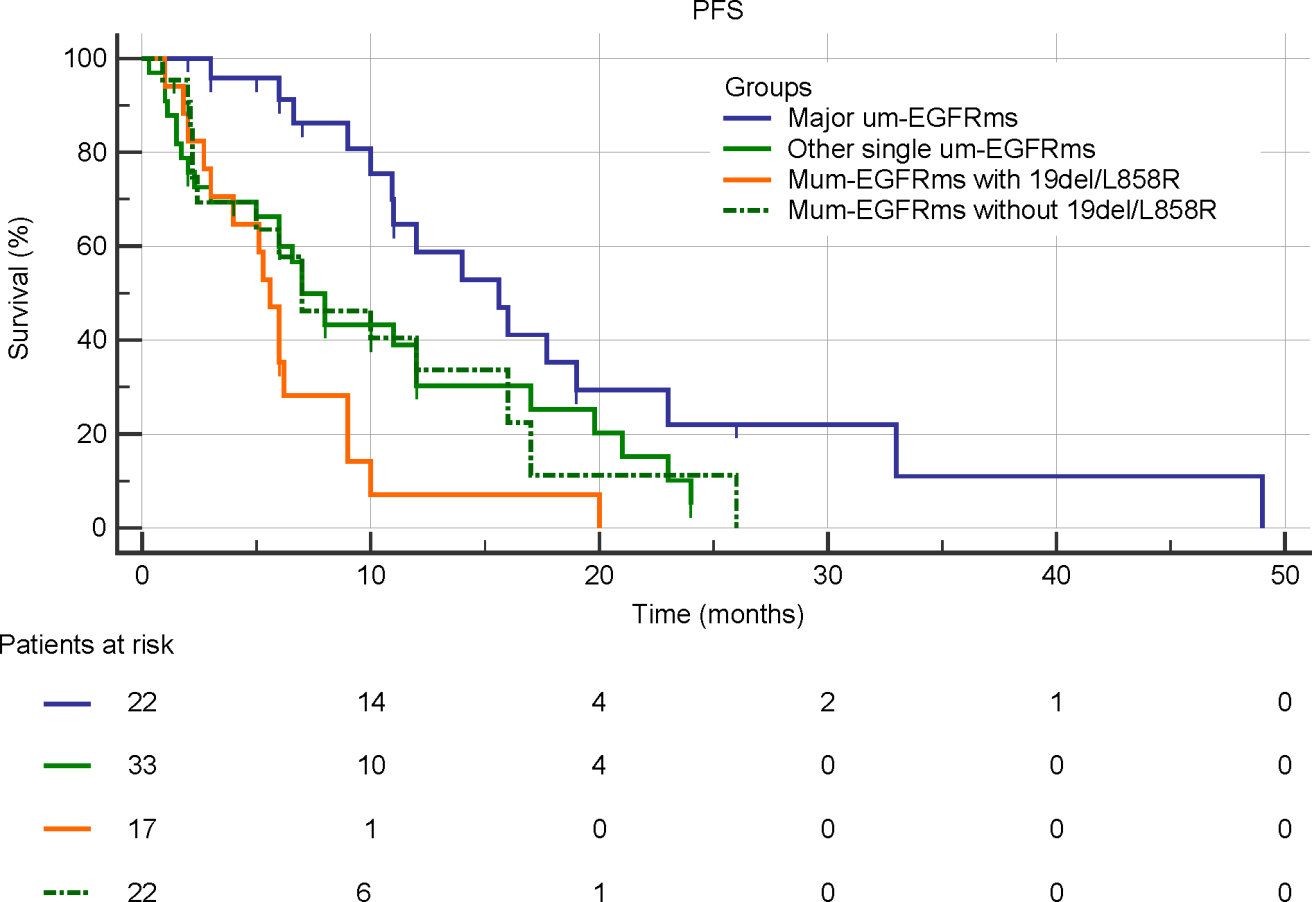
Kaplan-Meier curves for PFS in subgroup analysis according to mutation patterns.

Tumor response and PFS of each individual patient as well as overall tumor response rate and median PFS for each group were shown in **Figure 6**. The baseline characteristics of the patients in the four subgroups are shown in the **Supplement Table 3**. Subgroup analysis showed that patients in group A had the best efficacy and prognosis, with an ORR of 74.1% and an mPFS of 15.6 months. In contrast, the treatment efficacy was poorer in patients in Groups C, with ORRs of 23.5%, and corresponding mPFS times of 5.6 months, respectively. While, the efficacy and prognosis of groups B and D were similar, with ORR of 54.5% and 50.0%, and corresponding mPFS were 7.0 months in both groups (**Figure 7**). When comparing the ORR and mPFS of patients in Group A to those of patients in Group C, there was a statistically significant difference (ORR, HR: 9.286, 95% CI: 2.260-38.150, p=0.002; mPFS, HR: 0.204, 95% CI: 0.094-0.442, p<0.001) (**Figure 7**). Kaplan–Meier curves also showed that the PFS was longest in group A and shortest in groups C, while moderate in groups B and C (**Figure 8**).

## Discussion

In this study, the clinicopathological characteristics of 99 NSCLC patients with um-EGFRm were investigated, and their correlation with the efficacy and prognosis following afatinib were analyzed. In generally, the ORR of patients with um-EGFRm was53.5%, and the median PFS was 9.0 month. For patients administered first-line afatinib treatment, the ORR and median PFS were 73.3% and 15.6 months, respectively, which were both superior to those of patients treated with second- and later-line treatments. Moreover, single um-EGFRm and first-line treatment was an independent predictor of favorable treatment response and longer PFS. Subgroup analysis indicated that patients harboring major um-EGFRm had a favorable response to afatinib treatment and prognoses benefit; in contrast, patients harboring um-EGFRm that comprising 19del/L858R had a poorer response to treatment and unfavorable prognoses.

EGFR-mutant NSCLC is a genetically heterogeneous disease that includes more than 200 different mutant subtypes (13, 15). Uncommon and common EGFR mutations have been demonstrated to have similar clinicopathological characteristics (15), but patients with um-EGFRms are less sensitive to first-generation EGFR-TKI therapy (15, 26). Patients with NSCLC harboring um-EGFRm had a poorer response, lower ORR and shorter PFS than those of patients with 19del/L858R after receiving first-generation EGFR-TKIs (13, 15, 19). Regarding afatinib treatment, A *post hoc* analysis of the LUX-Lung 2, 3 and 6 clinical trials revealed that patients with um-EGFRms other than T790M and ex20ins who received first-line afatinib had an ORR of 71.0% and a median PFS of 10.7 months (95% CI: 5.6-14.7) (14). Our results were consistent with those of this study; in the present study, a total of 50 patients were treated with first-line afatinib, and their ORR and median PFS were 72.0% and 12.0 months, respectively. Another study evaluated the clinical efficacy of afatinib in 315 patients with NSCLC carrying um-EGFRms in randomized clinical trials or real-world cases, and the results showed that patients treated with afatinib who harbored major um-EGFRms and harbored multiple um-EGFRms had an ORR of 60.0% and 77.1%, respectively, with a corresponding median time to treatment failure (TTF) of 10.8 months and 14.7 months, respectively (17). These findings suggest that afatinib has favorable activity in patients with um-EGFRms. The results of several real-world observational investigations are consistent with these clinical trial data and demonstrate that afatinib is more efficacious than first-generation EGFR-TKIs in patients with um-EGFRms (18–21). For example, a retrospective study showed that patients with um-EGFRms treated with afatinib had an ORR and disease control rate (DCR) of 75% and 100%, respectively, which were significantly higher than those of patients treated with gefitinib or erlotinib, who had an ORR and DCR of 40% and 80%, respectively. Additionally, afatinib treatment was associated with longer PFS (17.1 months vs. 5.5 months) (18). In another retrospective study of 125 patients with um-EGFRm, compared to those treated with gefitinib and erlotinib, patients treated with afatinib demonstrated a higher ORR (afatinib 78.9%, gefitinib 38.9%, erlotinib 20%, p =0.013), as well as longer PFS (afatinib 10.5, gefitinib 3, erlotinib 0.9 months, p= 0.013) (19). Other real-world research has also demonstrated that patients with um-EGFRm treated with afatinib have more favorable prognoses than those of patients receiving gefitinib or erlotinib (20, 21). Moreover, a recent phase II clinical study (KCSG-LU15-09) of small sample of NSCLC patients harboring um-EGFRm indicated that osimertinib, a third-generation EGFR-TKI, exhibited clinical activity in patients with um-EGFRms. A total of 36 patients were treated with osimertinib (22 as first-line, 11 as second-line, and 3 as third-line), resulting in an ORR of 50.0% (95% CI: 33%-67%) and a median PFS of 8.2 months (95% CI: 5.9-10.5 months) (27). Presently, the available clinical data on osimertinib treatment for patients with um-EGFRms are limited; only a few cases or case series have reported the efficacy of osimertinib in patients with certain types of um-EGFRms (28–30). In view of the above findings, we can deduce that the second-generation EGFR-TKI, afatinib, has higher clinical activity in patients with um-EGFRms than first- and third-generation EGFR-KTIs, and this is also supported by the results of preclinical studies that um-EGFRms have higher affinity and sensitivity to afatinib (22–25).

Data from clinical trials and real-world research reveal that patients with um-EGFRms exhibit inconsistent responses and survival outcomes following afatinib treatment, which are closely related to the mutation pattern and the cooccurring partner mutant genes (13, 15). Therefore, we performed subgroup analysis to investigate the differences in the treatment efficacy of afatinib among patients with different types of um-EGFRms and the prognoses of these patients to determine which, if any, potential subgroups of patients are more likely to benefit from afatinib treatment. Ex20ins is the third most common EGFR mutation, accounting for approximately 10-12% of all EGFR mutations (31, 32). However, due to steric hindrance at the drug binding pocket, most of the EGFR proteins harboring these mutations are relatively insensitive to EGFR-TKIs, including afatinib (32, 33). In the *post-hoc* analysis of the LUX-lung trials, 23 patients with ex20ins who were treated with afatinib had an ORR of 8.7% and a PFS of only 2.7 months, representing the lowest efficacy of afatinib treatment over other types of um-EGFRms (14). Another study reported a slightly higher efficacy of afatinib in patients with ex20ins, with an ORR of 23.4% and a median TTF of 4.2 months (17). A Spanish multicenter retrospective study also showed that the treatment efficacy of afatinib was significantly lower in patients carrying ex20ins than in patients with other types of mutations, with an ORR of 13.0% and a median OS of 10.7 month (34). Therefore, platinum-based combination chemotherapy, rather than afatinib, might be the preferred treatment option for patients with ex20ins. Of note, the FDA currently has approved amivantamab, an EGFR-MET bispecific antibody, for the treatment of patients with locally advanced or metastatic NSCLC patients harboring an EGFR ex20ins mutation who exhibit disease progression during or after platinum-based chemotherapy. In addition to ex20ins, the other most frequently um-EGFRms include G719X in exon 18(including G719A, G719C, G719D, and G719S and other variants), S768I in exon 20, and L861Q in exon 21, which are known as major um-EGFRms and have been reported to demonstrate sensitivity to afatinib in preclinical and clinical studies (14, 17, 22–25). In *post hoc* analysis of the LUX-lung trials, patients carrying G719X, S768I and L861Q had ORRs of 78%, 100% and 56% after afatinib treatment, respectively, with corresponding median PFS of 13.8, 14.7 and 8.2 months, respectively, which represents the best demonstrated efficacy of afatinib (14). The results of our study are consistent with this finding: the patients carrying major um-EGFRms had an ORR of 74.1% and a median PFS of 15.6 months. This result is also supported by the results of other clinical trials and real-world clinical data (17, 20, 35). Obviously, afatinib should be considered the preferred treatment option for NSCLC patients carrying major um-EGFRms. Single EGFR mutations other than ex20ins and the major um-EGFRms were classified as other single um-EGFRms in this study, and patients with these mutations showed moderate sensitivity to afatinib, with an ORR of 54.5% and a median PFS of 7.0 months. Afatinib showed activity against several mutation types in this class of mutations, such as E709X in exon 18, L747P in exon 19, L774X, R776X, and Q787Q in exon 20, and H833V and H835L in exon 21 (13, 36). However, due to the high heterogeneity of patients with this category of mutations, including different types of mutations and variants, and the low mutation frequency, the available clinical data on the efficacy of afatinib are limited, and conclusions regarding the overall efficacy of afatinib are not uniform. Thus, further studies are warranted.

In addition to single mutations, two or more different types of EGFR mutations may coexist in tumor cells, which accounts for approximately 4-14% of EGFR mutations. Previous studies have indicated that the efficacy of EGFR-TKIs in patients with multiple um-EGFRms may be affected by the sensitivity of the accompanying mutations (13). We found that patients with multiple um-EGFRms containing 19del/L858R had the worst prognoses, with an ORR of only 23.5% and an mPFS of 5.6 months. In contrast, patients with multiple um-EGFRms without 19del/L858R showed a higher sensitivity to afatinib, with an ORR of 50.0% and a median PFS of 7.0 months. This result is similar to the results of a previous retrospective study, in which patients with multiple um-EGFRms who did not harbor 19del/L858R had better PFS than patients with both 19del/L858R (19). This may be due to the presence of major um-EGFRms among patients who harboring multiple um-EGFRms that without 19del/L858R. For example, Yang et al. reported that in patients with multiple um-EGFRms containing a major um-EGFRms, the ORR was 78.3%, and the median duration of response (DoR) was 17.1 months after receiving afatinib (14). This result is consistent with those of preclinical studies concluding that afatinib has broader inhibition than first- and third-generation EGFR-TKIs for patients with multiple EGFR mutations, particularly those harboring major um-EGFRms (22–25). In this study, among the 22 patients in Group D, 14 had a major um-EGFRm, while in Group C, only one patient carried a major um-EGFRms. Additionally, among the patients in Group D, three patients also carried the T790M mutation, which is considered a mutation that promotes resistance to afatinib treatment (37, 38). Therefore, administering afatinib for the treatment of patients with multiple um-EGFRm might be considered an effective treatment option in some circumstances. However, given the wide heterogeneity of patients with multiple um-EGFRms and the limited clinical data available, clinicians should make prudent clinical decisions based on a thorough understanding of the sensitivity and resistance of known mutated genes, especially concurrent partner mutations.

There are some unavoidable limitations of this study. Firstly, this is a re-analysis based on published research. This may be affected by, such as, selection bias, publication bias, and other uncontrollable confounding factors. Secondly, due to the variability of the included articles, there were not enough data for comparison of drug toxicity and side effects. Thirdly, the biological source, platform and method for EGFR detection are unclear, which could have an impact on the consistency and rate of EGFR detection. In addition, due to the heterogeneity of the included literature, we were unable to determine the site of metastasis in each patient and therefore could not investigate the relationship between the sites of metastasis and the type of mutation. Therefore, a further, large-scale, randomized controlled clinical study is needed to validate our conclusions.

## Conclusion

In summary, as a special type of EGFR mutation, patients with um-EGFRms exhibit favorable but inconsistent responses and survival outcomes following afatinib treatment. Our findings suggest that NSCLC patients carrying um-EGFRms can be further classified into various mutation subgroups that exhibit different responses and survival outcomes following afatinib treatment, but this conclusion requires further clinical studies for verification.

## Data availability statement

The raw data supporting the conclusions of this article will be made available by the authors, without undue reservation.

## Ethics statement

The studies involving human participants were reviewed and approved by the Ethics Committee of Yantai Yuhuangding Hospital. The ethics committee waived the requirement of written informed consent for participation.

## Author contributions

Conceptualization, SH and ZM; Methodology, CSW and CJW; Software, ZM and CSW; Formal Analysis, CJW and KZ; Resources, CJW; Data Curation, WD; Writing-Original Draft Preparation, CSW and WD; Writing-Review and Editing, SH and CSW; Supervision, SH and CJW. All authors contributed to the article and approved the submitted version.

## Funding

This research is supported by the Yantai Science and Technology Bureau Support Grant/Science and Technology Innovation Development Project (2020MSGY085), the Natural Science Foundation of Shandong Province (No. ZR2020QH204), and Yantai Policy Guidance Project (2021YD023).

## Acknowledgments

This study is a re-analysis of published articles, the included articles are listed in the supplement table and we thank the authors of these articles for their works.

## Conflict of interest

The authors declare that the research was conducted in the absence of any commercial or financial relationships that could be construed as a potential conflict of interest.

## Publisher’s note

All claims expressed in this article are solely those of the authors and do not necessarily represent those of their affiliated organizations, or those of the publisher, the editors and the reviewers. Any product that may be evaluated in this article, or claim that may be made by its manufacturer, is not guaranteed or endorsed by the publisher.
