# Supplementary material for: Inconsistent clinical outcomes following afatinib treatment in NSCLC patients harboring uncommon epidermal growth factor receptor mutation
Source: Front Oncol. 2022 Nov 8;12:999606. doi: 10.3389/fonc.2022.999606 (PMC9680984; doi:10.3389/fonc.2022.999606)
Supplement: Supplementary file 1 [file DataSheet_1.pdf]

Supplement Table 1. The studies included for this pooled analysis

Study	Study type	years	No. of patients	References*
Chinese retrospective study	Single-Center China study in patients with uncommon EGFR L747P and L747S mutations	2019	3	[1]
Germany retrospective study	Single-Center Germany study in patients with uncommon EGFR mutations treated with afatinib	2019	2	[2]
Germany retrospective study	Single-Center Germany study in patients with rare or complex EGFR mutations treated with EGFR TKIs	2019	3	[3]
Chinese retrospective study	Single-Center China study in patients with uncommon EGFR mutations treated with afatinib	2018	5	[4]
Japanese retrospective study	Single-Center Japan study in patients with complex EGFR mutations treated with afatinib	2017	1	[5]
Chinese retrospective study	Single-Center China study in patients with uncommon EGFR mutations treated with afatinib	2020	3	[6]
Japanese retrospective study	Single-Center Japan study in patients with uncommon EGFR mutations	2019	3	[7]
India retrospective study	Single-Center study in patients with rare EGFR mutations	2021	4	[8]
Chinese retrospective study	Single-Center China study in patients with uncommon EGFR mutations treated with afatinib	2020	2	[9]
Chinese retrospective	Single-Center China study in patients with uncommon	2018	2	[10]

study	EGFR mutations treated with afatinib			
Italy retrospective study	Single-Center study in patients with uncommon EGFR mutations	2018	1	[11]
Chinese retrospective study	Multiple-Center China study in patients with EGFR Exon 20 Insertion Mutations treated with Afatinib	2021	8	[12]
Case reports/case series	Various(n=58)	2015-2022	62	[13-70]

*: References here refer to the articles included in this pooled-analysis, not the references cited in the manuscript, which are listed in the reference section of the manuscript.

References

1. Liang SK, Ko JC, Yang JC, Shih JY. Afatinib is effective in the treatment of lung adenocarcinoma with uncommon EGFR p.L747P and p.L747S mutations. Lung Cancer. 2019 Jul;133:103-09.
2. Martin J, Lehmann A, Klauschen F, Hummel M, Lenze D, Grohe C, et al. Clinical Impact of Rare and Compound Mutations of Epidermal Growth Factor Receptor in Patients With Non-Small-Cell Lung Cancer. Clin Lung Cancer. 2019 Sep;20(5):350-62 e4.
3. Kauffmann-Guerrero D, Huber RM, Reu S, Tufman A, Mertsch P, Syunyaeva Z, et al. NSCLC Patients Harbouring Rare or Complex EGFR Mutations Are More Often Smokers and Might Not Benefit from First-Line Tyrosine Kinase Inhibitor Therapy. Respiration. 2018;95(3):169-76.
4. Wang DD, Lee VH, Zhu G, Zou B, Ma L, Yan H. Selectivity profile of afatinib for EGFR-mutated non-small-cell lung cancer. Mol Biosyst. 2016 Apr 26;12(5):1552-63.
5. Ishii H, Azuma K, Sakai K, Naito Y, Matsuo N, Tokito T, et al. Determination of Somatic Mutations and Tumor Mutation Burden in Plasma by CAPP-Seq during Afatinib Treatment in NSCLC Patients Resistance to Osimertinib. Sci Rep. 2020 Jan 20;10(1):691.

6. Ma C, Zhang J, Tang D, Ye X, Li J, Mu N, et al. Tyrosine Kinase Inhibitors Could Be Effective Against Non-small Cell Lung Cancer Brain Metastases Harboring Uncommon EGFR Mutations. Front Oncol. 2020;10:224.
7. Ikemura S, Yasuda H, Matsumoto S, Kamada M, Hamamoto J, Masuzawa K, et al. Molecular dynamics simulation-guided drug sensitivity prediction for lung cancer with rare EGFR mutations. Proc Natl Acad Sci U S A. 2019 May 14;116(20):10025-30.
8. Mehta A, Vasudevan S. Rare epidermal growth factor receptor gene alterations in non-small cell lung cancer patients, tyrosine kinase inhibitor response and outcome analysis. Cancer Treat Res Commun. 2021 May 13;28:100398.
9. Lin YT, Tsai TH, Wu SG, Liu YN, Yu CJ, Shih JY. Complex EGFR mutations with secondary T790M mutation confer shorter osimertinib progression-free survival and overall survival in advanced non-small cell lung cancer. Lung Cancer. 2020 Jul;145:1-9.
10. Tsai MJ, Hung JY, Lee MH, Kuo CY, Tsai YC, Tsai YM, et al. Better Progression-Free Survival in Elderly Patients with Stage IV Lung Adenocarcinoma Harboring Uncommon Epidermal Growth Factor Receptor Mutations Treated with the First-line Tyrosine Kinase Inhibitors. Cancers (Basel). 2018 Nov 13;10(11).
11. Improta G, Zupa A, Natalicchio MI, Sisinni L, Marinaccio A, Bozza G, et al. Uncommon frame-shift exon 19 EGFR mutations are sensitive to EGFR tyrosine kinase inhibitors in non-small cell lung carcinoma. Med Oncol. 2018 Jan 31;35(3):28.
12. Wei Y, Jiang B, Liu S, Zhang Z, Fang W, Yang Y, et al. Afatinib as a Potential Therapeutic Option for Patients With NSCLC With EGFR G724S. JTO Clin Res Rep. 2021 Jul;2(7):100193.
13. Yamaguchi T, Hayashi H, Isogai S, Hayashi M, Uozu S, Goto Y, et al. Afatinib administration in a patient with non-small cell lung cancer harboring uncommon EGFR mutation G719A undergoing hemodialysis. Cancer Treatment Communications. 2015;4:169-71.
14. Yang X, Chen H, Zhang H, Duan J, An T, Zhao J, et al. Effectiveness of Tyrosine Kinase Inhibitors on Uncommon Epidermal Growth Factor Receptor Mutations in Non-small Cell Lung Cancer. Zhongguo Fei Ai Za Zhi. 2015 Aug;18(8):493-9.
15. Frega S, Conte P, Fassan M, Polo V, Pasello G. A Triple Rare E709K and L833V/H835L EGFR Mutation Responsive to an Irreversible Pan-HER Inhibitor: A Case Report of Lung Adenocarcinoma Treated with Afatinib. J Thorac Oncol. 2016 May;11(5):e63-e64.

16. Tanizaki J, Banno E, Togashi Y, Hayashi H, Sakai K, Takeda M, et al. Case report Durable response to afatinib in a patient with lung cancer harboring two uncommon mutations of EGFR and a KRAS mutation. Lung Cancer. 2016;101:11-15.
17. Agatsuma N, Yasuda Y, Ozasa H. Malignant Pleural Mesothelioma Harboring Both G719C and S768I Mutations of EGFR Successfully Treated with Afatinib. J Thorac Oncol. 2017 Sep;12(9):e141-e43.
18. Frega S, Lorenzi M, Fassan M, Indraccolo S, Calabrese F, Favaretto A, et al. Clinical features and treatment outcome of non-small cell lung cancer (NSCLC) patients with uncommon or complex epidermal growth factor receptor (EGFR) mutations. Oncotarget. 2017;8(20):32626-38.
19. Furuya T, Shimada J, Okada S, Tsunezuka H, Kato D, Inoue M. Successful treatment with afatinib for pancreatic metastasis of lung adenocarcinoma: a case report. J Thorac Dis. 2017 Oct;9(10):E890-E93.
20. Ibrahim U, Saqib A, Atallah JP. EGFR exon 18 delE709_T710insD mutated stage IV lung adenocarcinoma with response to afatinib. Lung Cancer. 2017 Jun;108:45-47.
21. Nakamura D, Miura K, Kumeda H, Agatsuma H, Hyogotani A, Hamanaka K, et al. Successful Resection of G719X-Positive Pleomorphic Carcinoma after Afatinib Treatment. Case Rep Oncol. 2017 Sep-Dec;10(3):1035-40.
22. Russo A, Franchina T, Ricciardi GRR, Adamo V. Rapid Acquisition of T790M Mutation after Treatment with Afatinib in an NSCLC Patient Harboring EGFR Exon 20 S768I Mutation. J Thorac Oncol. 2017 Jan;12(1):e6-e8.
23. Shen YC, Tseng GC, Tu CY, Chen WC, Liao WC, Chen WC, et al. Comparing the effects of afatinib with gefitinib or Erlotinib in patients with advanced-stage lung adenocarcinoma harboring non-classical epidermal growth factor receptor mutations. Lung Cancer. 2017 Aug;110:56-62.
24. Tamiya M, Shiroyama T, Nishihara T, Nishida T, Hayama M, Tanaka A, et al. Afatinib successfully treated leptomeningeal metastasis during erlotinib treatment in a patient with EGFR-mutant (Exon18:G719S) lung adenocarcinoma as a second-line chemotherapy. Asia Pac J Clin Oncol. 2017 Oct;13(5):e531-e33.

25. Velcheti V, Khunger M, Abazeed ME. Novel EGFR Exon 18 (G721R) Mutation in a Patient with Non-Small Cell Lung Carcinoma with Lack of Response to Afatinib. J Thorac Oncol. 2017 Feb;12(2):e16-e18.
26. Zhu X, Bai Q, Lu Y, Qi P, Ding J, Wang J, et al. Response to Tyrosine Kinase Inhibitors in Lung Adenocarcinoma with the Rare Epidermal Growth Factor Receptor Mutation S768I a Retrospective Analysis and Literature Review. Target Oncol. 2017;12(1):81-88.
27. Coupkova H, Vyzula R. Afatinib in the Treatment of Advanced Non-Small Cell Lung Cancer with Rare EGFR (in exon 18-T179X) Mutation - a Case Report. Klin Onkol. 2018 Fall;31(5):380-83.
28. Duan H, Peng Y, Cui H, Qiu Y, Li Q, Zhang J, et al. Effectiveness of afatinib after ineffectiveness of gefitinib in an advanced lung adenocarcinoma patient with a single EGFR exon 20 S768I mutation: a case report. Onco Targets Ther. 2018;11:2303-09.
29. Galli G, Corrao G, Imbimbo M, Proto C, Signorelli D, Ganzinelli M, et al. Uncommon mutations in epidermal growth factor receptor and response to first and second generation tyrosine kinase inhibitors: A case series and literature review. Lung Cancer. 2018 Jan;115:135-42.
30. Nasu S, Shiroyama T, Morita S, Takata S, Takada H, Masuhiro K, et al. Osimertinib Treatment Was Unsuccessful for Lung Adenocarcinoma with G719S, S768I, and T790M Mutations. Intern Med. 2018 Dec 15;57(24):3643-45.
31. Niogret J, Coudert B, Boidot R. Primary Resistance to Afatinib in a Patient with Lung Adenocarcinoma Harboring Uncommon EGFR Mutations: S768I and V769L. J Thorac Oncol. 2018 Jul;13(7):e113.
32. Qin BD, Jiao XD, Yuan LY, Liu K, Wang Z, Qin WX, et al. The effectiveness of afatinib and osimertinib in a Chinese patient with advanced lung adenocarcinoma harboring a rare triple EGFR mutation (R670W/H835L/L833V): a case report and literature review. Onco Targets Ther. 2018;11:4739-45.
33. van Kempen LC, Wang H, Aguirre ML, Spatz A, Kasymjanova G, Vilacha JF, et al. Afatinib in Osimertinib-Resistant EGFR ex19del/T790M/P794L Mutated NSCLC. J Thorac Oncol. 2018 Sep;13(9):e161-e63.
34. Watanabe M, Oizumi S, Kiuchi S, Yamada N, Yokouchi H, Fukumoto S, et al. The Effectiveness of Afatinib in a Patient with Advanced Lung Adenocarcinoma Harboring Rare G719X and S768I Mutations. Intern Med. 2018 Apr 1;57(7):993-96.

35. Zeng L, Zhang Y, Yang N. EGFR exon 18 DelE709_T710insD as an Acquired Resistance Mechanism to Afatinib in an Advanced EGFR exon 18 E709H Lung Adenocarcinoma. J Thorac Oncol. 2018 Jun;13(6):e93-e95.
36. Zhou T, Zhou X, Li P, Qi C, Ling Y. EGFR L747P mutation in one lung adenocarcinoma patient responded to afatinib treatment: a case report. J Thorac Dis. 2018 Dec;10(12):E802-E05.
37. An N, Wang H, Zhu H, Yan W, Jing W, Kong L, et al. Great efficacy of afatinib on a patient with lung adenocarcinoma harboring uncommon EGFR delE709_T710insD mutations: a case report. Onco Targets Ther. 2019;12:7399-404.
38. Del Re M, Rofi E, Cappelli C, Puppo G, Crucitta S, Valeggi S, et al. The increase in activating EGFR mutation in plasma is an early biomarker to monitor response to osimertinib: a case report. BMC Cancer. 2019 Apr 30;19(1):410.
39. Iwamoto Y, Ichihara E, Hara N, Nakasuka T, Ando C, Umeno T, et al. Efficacy of afatinib treatment for lung adenocarcinoma harboring exon 18 delE709_T710insD mutation. Jpn J Clin Oncol. 2019 Aug 1;49(8):786-88.
40. Liu J, Jin B, Su H, Qu X, Liu Y. Afatinib helped overcome subsequent resistance to osimertinib in a patient with NSCLC having leptomeningeal metastasis baring acquired EGFR L718Q mutation: a case report. BMC Cancer. 2019 Jul 17;19(1):702.
41. Ma C, Huang C, Tang D, Ye X, Li Z, Liu R, et al. Afatinib for Advanced Non-small Cell Lung Cancer in a Case With an Uncommon Epidermal Growth Factor Receptor Mutation (G719A) Identified in the Cerebrospinal Fluid. Front Oncol. 2019;9:628.
42. Taniguchi Y, Yamamoto M, Ikushima H, Ohara S, Takeshima H, Sakatani T, et al. Successful Treatment of Afatinib-Refractory Non-Small Cell Lung Cancer with Uncommon Complex EGFR Mutations Using Pembrolizumab: A Case Report. Case Rep Oncol. 2019 May-Aug;12(2):564-67.
43. Zhang H, Shao YW, Xia Y. Responsiveness to Full-Dose Afatinib in a Patient With Lung Adenocarcinoma Harboring EGFR S768I and V769L Mutations. J Thorac Oncol. 2019 Feb;14(2):e25-e27.
44. Zhu N, Dong C, Weng S, Yuan Y, Yuan Y. A Patient of Advanced NSCLC with a New EGFR Exon 19 Insertion Mutation and its Response to EGFR-TKIs. J Coll Physicians Surg Pak. 2019;29(12):S126-S28.

45. Iida Y, Kumasawa F, Shimizu T, Shintani Y, Takahashi N, Gon Y. Successful treatment of an elderly patient with an uncommon L861Q epidermal growth factor receptor mutation with low-dose afatinib: A case report. Thorac Cancer. 2020 Feb;11(2):447-50.
46. Long X, Qin T, Lin J. Great Efficacy of Afatinib in a Patient with Lung Adenocarcinoma Harboring EGFR L833V/H835L Mutations: A Case Report. Onco Targets Ther. 2020;13:10689-92.
47. Ma C, Liu M, Mu N, Li J, Li L, Jiang R. Efficacy of afatinib for pulmonary adenocarcinoma with leptomeningeal metastases harboring an epidermal growth factor receptor complex mutation (exon 19del+K754E): A case report. Medicine (Baltimore). 2020 Oct 23;99(43):e22851.
48. Ma C, Wang S, Mu N, Li J, Liu M, Li L, et al. Effective Treatment With Afatinib of Lung Adenocarcinoma With Leptomeningeal Metastasis Harboring the Exon 18 p.G719A Mutation in the EGFR Gene Was Detected in Cerebrospinal Fluid: A Case Report. Front Oncol. 2020;10:1635.
49. Yang Y, Zhang X, Wang R, Qin J, Wang J, Li Z, et al. Osimertinib Resistance With a Novel EGFR L858R/A859S/Y891D Triple Mutation in a Patient With Non-Small Cell Lung Cancer: A Case Report. Front Oncol. 2020;10:542277.
50. Zhang C, Lin L, Zuo R, Wang Y, Chen P. Response to tyrosine kinase inhibitors in lung adenocarcinoma with the rare epidermal growth factor receptor mutation S768I and G724S: A case report and literature review. Thorac Cancer. 2020 Sep;11(9):2743-48.
51. Zochbauer-Muller S, Kaserer B, Prosch H, Cseh A, Solca F, Bauer MJ, et al. Case Report: Afatinib Treatment in a Patient With NSCLC Harboring a Rare EGFR Exon 20 Mutation. Front Oncol. 2020;10:593852.
52. Bi H, Ren D, Wu J, Ding X, Guo C, Miura S, et al. Lung squamous cell carcinoma with rare epidermal growth factor receptor mutation G719X: a case report and literature review. Ann Transl Med. 2021 Dec;9(24):1805.
53. Guo T, Zhu L, Li W, Lin R, Ding Y, Kang Q, et al. Two cases of non-small cell lung cancer patients with somatic or germline EGFR R776H mutation. Lung Cancer. 2021 Nov;161:94-97.
54. He SY, Lin QF, Chen J, Yu GP, Zhang JL, Shen D. Efficacy of afatinib in a patient with rare EGFR (G724S/R776H) mutations and amplification in lung adenocarcinoma: A case report. World J Clin Cases. 2021 Feb 26;9(6):1329-35.

55. Jelli B, Taton O, D'Haene N, Remmelink M, Mekinda Z. Complete Response to Afatinib of an EGFR Exon 18 delE709_T710insD-Mutated Stage IV Lung Adenocarcinoma. Eur J Case Rep Intern Med. 2021;8(8):002749.
56. Liu X, Ma B, Li T, Zhao L. Case Report: Afatinib-Induced Interstitial Pneumonia: Experiences and Lessons From Two Patients. Front Pharmacol. 2021;12:698447.
57. Longo V, Catino A, Montrone M, Pizzutilo P, Pesola F, Marech I, et al. Successful treatment of triple EGFR mutation T785A/L861Q/H297_E298 with afatinib. Thorac Cancer. 2021 Jul;12(13):2031-34.
58. Minari R, Leonetti A, Gnetti L, Zielli T, Ventura L, Bottarelli L, et al. Afatinib therapy in case of EGFR G724S emergence as resistance mechanism to osimertinib. Anticancer Drugs. 2021 Mar 5.
59. Morita A, Hosokawa S, Yamada K, Umeno T, Kano H, Kayatani H, et al. Dacomitinib as a retreatment for advanced non-small cell lung cancer patient with an uncommon EGFR mutation. Thorac Cancer. 2021 Apr;12(8):1248-51.
60. Qu F, Wu S, Dong H, Yan X. An elderly advanced non-small cell lung cancer patient harboring rare epidermal growth factor receptor mutations L861R benefited from afatinib: A case report. Medicine (Baltimore). 2021 Nov 12;100(45):e27614.
61. Shijubou N, Sumi T, Kamada K, Sawai T, Yamada Y, Nakata H, et al. Long-term response to afatinib in an elderly patient with uncommon epidermal growth factor receptor mutation-positive lung adenocarcinoma. Thorac Cancer. 2021 Mar;12(6):989-92.
62. Tamiya M, Kunimasa K, Nishino K, Matsumoto S, Kawachi H, Kuno K, et al. Successful treatment of an osimertinib-resistant lung adenocarcinoma with an exon 18 EGFR mutation (G719S) with afatinib plus bevacizumab. Invest New Drugs. 2021 Feb;39(1):232-36.
63. Tamura T, Kawakado K, Makimoto G, Nakanishi M, Kuyama S. Limited effect of afatinib in a non-small cell lung cancer patient harboring an epidermal growth factor receptor K860I missense mutation: A case report. Thorac Cancer. 2021 Jun;12(11):1770-74.
64. Van Acker L, Stevens D, Vermaelen K, Surmont V. Afatinib for the treatment of advanced non-small-cell lung cancer harboring an epidermal growth factor receptor exon 18 E709_T710delinsD mutation: a case report. J Med Case Rep. 2021 Nov 22;15(1):562.
65. Wei Y, Cui Y, Guo Y, Li L, Zeng L. A Lung Adenocarcinoma Patient With a Rare EGFR E709_T710delinsD Mutation Showed a Good

Response to Afatinib Treatment: A Case Report and Literature Review. Front Oncol. 2021;11:700345.
66. Xiang C, Zhang W, Xiong LW, Cai XW, Teng HH, Zhao RY, et al. EGFR Thr790Leu as a Potential Resistance Mechanism to First-Generation EGFR Tyrosine Kinase Inhibitor May Respond to Osimertinib in Patients With Lung Adenocarcinoma. JTO Clin Res Rep. 2021 Jul;2(7):100185.
67. Yu N, Xu Y, Wang X, Sun C, Qiu S, Guo Y, et al. Successful Treatment of Afatinib Reversing Epidermal Growth Factor Receptor Exon19Deletion/G724S Mutation Resistance Guided by Protein-Drug Docking. Oncologist. 2021 Nov;26(11):e1903-e08.
68. Zhang Y, Shen JQ, Shao L, Chen Y, Lei L, Wang JL. Non-small-cell lung cancer with epidermal growth factor receptor L861Q-L833F compound mutation benefits from both afatinib and osimertinib: A case report. World J Clin Cases. 2021 Sep 26;9(27):8220-25.
69. Zhao Y, Zhai L, Deng L, Halmos B, Cheng H. Efficacy of Osimertinib in Afatinib-resistant Lung Cancer Harboring Uncommon EGFR Mutations: Case Report and Literature Review. Clin Lung Cancer. 2021 May;22(3):e466-e69.
70. Huang Q, Chen C, Hu S, Wu H, Yu D, Zhu X, et al. Long-term survival in a patient with advanced lung adenocarcinoma harboring synchronous EGFR exon 18 G719A and BRAF V600E mutations and treated with afatinib: a case report. Anticancer Drugs. 2022 Jan 1;33(1):e730-e33.

Supplement Table 2 Composition of un-common EGFR mutations

EGFR mutation types	Number	Percentage (%)
G719X	14	14.1
18 del	8	8.1
19 ins	7	7.1
L861Q	7	7.1
19 del/G724S	6	6.1
S768I	6	6.1
G719X/S768I	3	3.0
L747P	3	3.0
E709K/L833V/H835L	2	2.0
G724S/S768I	2	2.0
L833V/H835L	2	2.0
19 del/L747S	1	1.0
19 del/T790M/C797S	1	1.0
19 del/T790M/L833V	1	1.0
19 ins/ K754E	1	1.0
19del/T790M/P794L	1	1.0
A859T	1	1.0
G719X/L861Q	1	1.0
G719X/R776C	1	1.0
G719X/S781I	1	1.0
G721R	1	1.0
G724S	1	1.0
G724S/N771Dup	1	1.0
G724S/R776H	1	1.0
G724S/S761I	1	1.0
G857V	1	1.0
I706T	1	1.0
I740V	1	1.0
K716E	1	1.0
K860I	1	1.0
L730R	1	1.0
L833F/L861Q	1	1.0
L858R/A859S	1	1.0
L858R/I759M	1	1.0

L858R/L718Q	1	1.0
L858R/L833V	1	1.0
L858R/S768I	1	1.0
L858R/T790L	1	1.0
L858R/V834L	1	1.0
L861I/S720F	1	1.0
L861R	1	1.0
N771Y	1	1.0
P733Q	1	1.0
R670W/L833V/H835L	1	1.0
R776H/L861Q	1	1.0
S768I/V774M	1	1.0
S861I	1	1.0
T785A/L861Q	1	1.0
T790M/C797S	1	1.0
V769L	1	1.0
Total	99	100.0

Supplement Table 3. Baseline characteristics of subgroups

Characteristics	Total (n=99)	Major um-EGFRms[a] (n=27)	Other single um-EGFRms (n=33)	Mum-EGFRms with 19del/L858R[b] (n=17)	Mum-EGFRms without 19del/L858R[c] (n=22)
Age					
<60	54	15	19	11	9
≥60	45	12	14	6	13
Gender					
Male	53	13	18	8	14
Female	46	14	15	9	8
Ethnicity					
Asian	66	20	17	11	18
Non-Asian	33	7	16	6	4
Smoking					
Yes	39	11	12	8	8
No	60	16	21	9	14
Stage					
III	5	1	2	1	1
IV	94	26	31	16	21
Mutation number					
Single	60	27	33	0	0

Multiple	39	0	0	17	22
Afatinib lines					
1 L	45	17	14	3	11
≥2 L	54	10	19	14	11

a: L861Q, G719X, and S768I;

b: Multiple EGFR mutations that with 19del/L858R;

c: Multiple EGFR mutations that without 19del/L858R.
